# Conformational Changes in Proteins Caused by High-Pressure Homogenization Promote Nanoparticle Formation in Natural Bone Aqueous Suspension

**DOI:** 10.3390/foods11182869

**Published:** 2022-09-16

**Authors:** Xue Li, Zhifei He, Jingbing Xu, Chang Su, Xu Xiao, Ling Zhang, Huanhuan Zhang, Hongjun Li

**Affiliations:** 1 College of Food Science, Southwest University, Chongqing 400715, China; 2 Agricultural Product Processing Institute, Chongqing Academy of Agricultural Science, Chongqing 401329, China; 3 Chongqing Key Laboratory of Specialty Food Co-Built by Sichuan and Chongqing, Chongqing 400715, China; 4 Chongqing Institute for Food and Drug Control, Chongqing 401121, China

**Keywords:** nanoprocessing, high-pressure homogenization (HPH), rabbit bone, collagen, peptide calcium

## Abstract

As a natural calcium resource, animal bone needs to be miniaturized to the nanoscale to improve palatability and absorption capacity. To explore the mechanism of high-pressure homogenization (HPH) in preparing natural bone aqueous nanosuspensions, the relationships between the changes in protein conformation, solubility and quality characteristics of rabbit bone aqueous suspensions (RBAS) prepared by different HPH cycles were studied. The results showed that the improvements in particle size, stability and calcium solubility of RBASs could be mainly attributed to the improvement of protein solubility induced by the changes in protein conformation. HPH treatment led to the denaturation and degradation of protein in rabbit bone, generating soluble peptides and improving the stability of the suspensions by enhancing the surface charge of the particles. When collagen as the main protein was partially degraded, the hydroxyapatite in the bone was crushed into tiny particles. The increase in the particle-specific surface area led to the release of calcium ions, which chelated with the peptides to produce peptide calcium. However, excessive HPH treatment caused the production of protein macromolecular aggregates and affected the quality of RBASs. This study is helpful to promote the application of HPH technology in animal bone nanoprocessing.

## 1. Introduction

Animal bone is a meat processing byproduct that has great potential. On a dry-fat-free basis, animal bones are rich in minerals (50–70%, *w*/*w*) and protein (30–50%, *w*/*w*), of which calcium is as high as 20–30% (*w*/*w*) [1,2]. Type Ι collagen and hydroxyapatite [HAP, Ca_10_ (PO_4_) _6_(OH)_2_] are the main components of proteins and minerals in animal bone. A network structure composed of collagen is embedded with HAP to form a hard bone structure [3,4,5]. The highly ordered structure and the hydration layer formed outside the HAP protect the calcium compounds from direct contact with water, with the result that the calcium in animal bone is difficult to dissolve in water, acidic and alkaline solutions at normal temperatures [6]. Due to its large molecular weight and unique triple helix structure, collagen is very stable and difficult to digest and absorb. However, collagen peptides produced after collagen hydrolysis have extremely high nutritional value and physiological regulation function [7,8,9] and are very popular in the consumer market. If animal bone could be properly processed and developed into functional food by improving the grainy texture and the absorption of nutrients, it would not only bring great economic benefits and reduce the burden on the environment [10] but also promote the sustainable and green development of the meat industry.

In recent years, nanomachining technology has been studied in animal bone processing. By using the strong mechanical force generated by planetary ball milling, high-energy wet ball milling and high-pressure microfluidization, bone particles can be reduced to the nanoscale (<1000 nm) [2,11,12,13,14]. Nanoprocessing not only improved the particle characteristics of animal bone but also allowed better applications in gel food [10,11,15]. Moreover, due to the increase in specific surface area and the change in the crystal structure, calcium was released from HAP, which significantly improved the bioabsorption of calcium; these improvements were more significant than the reduction of bone particles to the micron scale [2,3,10,14]. Nanotechnology has great potential in the processing and utilization of animal bone.

The network structure composed of collagen fibers constitutes the toughness of bone [4], which is also the main factor preventing bone crushing. In a previous study, a HAP aqueous suspension (5%, *w*/*w*) was found to be easy to crush. The average particle size [Dx (50)] could be reduced from 80 μm to 4 μm only by using a high-speed shear emulsifying machine (Ultraturraxt25, IKA, Staufen, Germany) at 2800 rpm for 2 min. Under the same conditions, the particle size of the rabbit bone suspension (30 μm) did not change significantly and could be reduced only by ball milling or high-pressure homogenization (HPH). Compared with high-energy wet ball milling, the efficiency of HPH nanoprocessing is extremely high and can reduce the particle size to the nanoscale in a short time (<10 min), while high-energy wet ball milling usually takes 5 h or more [3,10,13]. This may be because HPH equipment can simultaneously apply high-speed shear, pressure gradient, high-speed collision and other forces to the raw materials, and these treatment conditions are usually very severe [16,17,18,19]. Iordache and Jelen [20] found that HPH could increase the solubility of heat-denatured whey protein, while Chen et al. [21] found that HPH could promote the dissolution of chicken breast myofibrillar proteins in water. Therefore, HPH treatment leads to the dissolution of collagen, which may be the reason why HPH can efficiently prepare bone aqueous nanosuspensions. However, research in this area is still rare, and most of the research has focused on the improvement of calcium solubility in animal bone by nanoprocessing [3,10,11,13,14].

Due to its advantages of high calcium, high protein and ease of crushing, rabbit bone is a suitable material for nanoprocessing [2]. Moreover, rabbit meat is popular worldwide as functional meat [22]; according to FAO statistics, the global rabbit meat production in 2020 was approximately 0.9 million tons [23]. With the rapid development of the rabbit industry, the utilization of rabbit bone and other byproducts cannot be ignored. Therefore, to explain the main mechanism by which HPH produces nanobone particles, this study used rabbit bone as an example. Micron-scale rabbit bone (MRB) and water were used as raw materials, and the liquid rabbit nanobone was prepared efficiently by using the physical means of HPH technology. At the same time, the relationships between the particle size, stability, solubility and protein conformation changes in rabbit bone aqueous suspensions (RBAS) were investigated. This information will provide a theoretical basis for the application of HPH technology in animal bone nanoprocessing.

## 2. Materials and Methods

### 2.1. Materials

**Figure 1:**
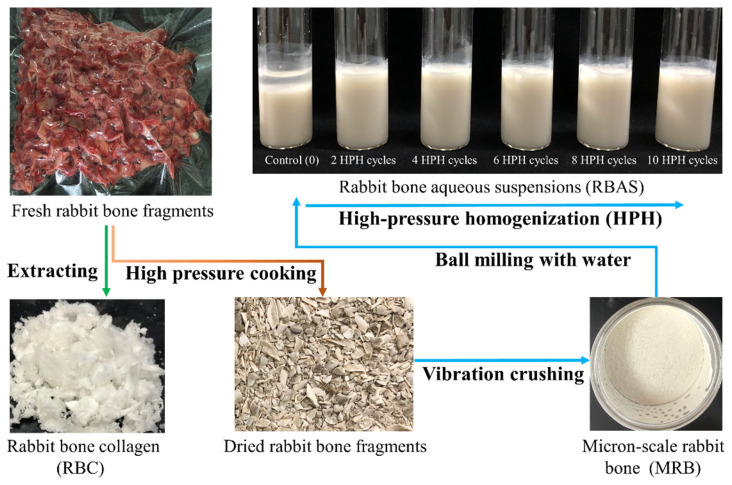
Schematic diagram of samples’ processing and their appearance. Fresh rabbit bone fragments were vacuum-packed. Rabbit bone collagen (RBC) was prepared by the acid dissolution method and freeze-dried in a vacuum. Micron-scale rabbit bone (MRB) was photographed in the receiving box of the vibratory crusher. The photo of rabbit bone aqueous suspensions (RBAS) was taken within 1 h after different high-pressure homogenization (HPH) cycles.

One hundred and fifty fresh HYLA rabbit carcasses (male, 72 days old, 2.2–2.6 kg per live rabbit) used in the experiment were purchased from the local slaughterhouse (Yubei, Chongqing, China) and transported back to the laboratory in a chilled box (approximately 8 °C) within 6 h. Then, the main limb bones (scapula, humerus, forearm, ilium, femur, calf) were separated and cleaned of muscle, fat and tendons within 4 h. Finally, all bones were ground and mixed through a PG-230 bone crusher (Yongchuang, Zhengzhou, China) and vacuum packaged within 2 h, and then frozen at −21 °C until use. Collagen from a small portion of the bones was acid-extracted (rabbit bone collagen, RBC) according to the method of Li et al. [24], and the remaining part was used to prepare MRB, which was the raw material for producing RBASs, as presented in Figure 1.

Synthetic HAP (H875582, 80 μm) was purchased from Macklin Reagent (Shanghai, China). Type I collagen standard (C8060, from bovine Achilles tendon) was purchased from Solarbio Technology Co., Ltd. (Beijing, China). All other chemical reagents used were analytical grade or higher and obtained from Chron Chemical Co., Ltd. (Chengdu, China).

### 2.2. RBAS Preparation

First, MRB was prepared according to the process described by Li et al. [2] with some modifications. Frozen bone shards were thawed at room temperature (26 °C) and then rinsed three times to remove blood and fat. Drained bone fragments were autoclaved at 120 °C and 0.1 MPa for 2 h by an LDZM-80KCS high-pressure cooker (Shenan, Shanghai, China). The softened bones were removed and rinsed with tap water to remove the oil and residual meat on the surface, and then the bone residues were dried in a DHG-9053 air blower drying oven (Sanfa, Shanghai, China) at 60 °C to constant weight. The dried bone pieces were crushed with a BJ-800A grinder (Baijie, Huzhou, China) and passed through a 60-mesh screen. For degreasing, the bone powder was mixed with ethyl acetate (1:5.3, *w*/*w*) and placed into a 40 kHz KQ-600DB ultrasonic machine (Kunshan Ultrasonic Instrument Co., Ltd., Kunshan, China) for ultrasonic extraction (30 °C, 480 W, 24 min). After that, the lipid-extracted bone was centrifuged at 4000× *g* for 10 min, and the precipitate was dried in a fume hood. Next, the dried, defatted bone powder was mixed with ultrapure water (1:3, *w*/*w*), stirred for 1 min and allowed to stand for 2 min. Then, the upper liquid was poured, and the red plasma protein on the surface was scraped off with a medicine spoon. To give the bone powder a better color, this operation was repeated three times to remove most of the plasma protein, and the remaining bone residue was dried at 60 °C to constant weight. Finally, the bone residue was crushed to the micron scale by an SYFM-8II vibrating microgrinder (Songyue, Jinan, China) with suitable parameters (4 °C, 5 mm vibration amplitude, 10 min), which yield MRB.

To obtain a suspension with an appropriate particle size, MRB was mixed with ultrapure water (1:3, *w*/*w*) and ground for 2 h by using an XQM-0.4 planetary ball mill (Tianchuang, Changsha, China). After that, the suspension was further adjusted to 5% (*w*/*w*) with ultrapure water. Thus, the control group was acquired and was stirred with a glass rod and immediately poured into the hopper of a dynamic high-pressure microfluidization machine (M-110EH-30, MFIC, Newton, MA, USA) equipped with a DC-2006 condenser (4 °C, Shunma, Nanjing, China) for HPH treatment. A series of control groups were homogenized at 15,000 PSI (103 MPa) for zero, two, four, six, eight and ten cycles to prepare RBASs, and they were rapidly divided into two portions: one portion was used for the determination of physiochemical characteristics, and the other portion was centrifuged at 4000× *g* for 30 min, and the supernatants were aspirated carefully to determine the solubility-related indicators. Finally, all samples were refrigerated at 4 °C until use.

### 2.3. Chemical Composition Analysis

#### 2.3.1. Proximate

The crude fat, moisture, crude protein, and ash contents of the MRB were determined by the standard A.O.A.C. method (960.39, 934.01, 928.08 and 920.153) [25], and the soluble component was based on the gelatin extraction method described by Gómez-Guillén et al. [26] with minor modifications. MRB with a certain mass (M_1_) was transferred to a centrifuge tube, mixed with ultrapure water (1:3, *w*/*w*) and then placed on a constant temperature shaker (Maxq4000, Thermo Fisher, Marietta, OH, USA) at 45 °C and 200 rpm for 1.5 h. Next, the suspension was centrifuged at 4000× *g* for 30 min, and the supernatant was poured into a drying dish (M_0_) and weighed (M_2_) after drying at 105 °C to constant weight. The precipitate was removed and dried at 60 °C until use. The soluble component of MRB was calculated according to Equation (1).

Soluble component (%) = (M_2_ − M_0_)/M_1_ × 100% (1)

The total protein, soluble protein and soluble peptides of the bone suspension were also determined according to the Kjeldahl method (928.08) of A.O.A.C. [25], while the soluble peptides were obtained by removing macromolecular proteins with trichloroacetic acid (TCA). The supernatant of the bone suspension was homogenized with the same volume of 20% (*w*/*w*) TCA for 1 min. After standing for 1 h, the mixed liquid was centrifuged at 4000× *g* for 30 min, and the supernatant was filtered and fixed to a constant volume for Kjeldahl nitrogen determination.

#### 2.3.2. Amino Acid Composition

The composition of amino acids was implemented by an amino acid analyzer (S433D, Sykam, Munich, Bavaria, Germany) equipped with a lithium system [2]. The solution preparation, sample pretreatment and procedures were carried out as outlined by Sykam Corp. The total amino acid contents of the supernatants of RBASs and the MRB were determined, while the protein impurities were removed by sulfosalicylic acid crystals (0.18 g in 1 mL solution) in the determination of free amino acids in RBAS supernatants.

#### 2.3.3. Elemental Analysis

The elemental composition was measured by inductively coupled plasma-atomic emission spectrometry (iCAP 6000, Thermo Fisher, Cambridge, MA, USA) according to Ersoy and Özeren [27]. For MRB, the contents of calcium, magnesium, phosphorus and zinc were measured, while for the supernatants of RBASs and peptide calcium, only the calcium content was measured.

#### 2.3.4. Molecular Weight Distribution of Peptides

The contents of peptides with different molecular weights were determined by the method of Li et al. [3], with some modifications. In each HPH treatment group, 2 mL of bone suspension supernatant was taken and centrifuged at 4000× *g* for 60 min in a series of centrifugal ultrafiltration tubes (Amicon Ultra-4, Millipore, Ireland), which consisted of cellulose membranes with different molecular weight cutoffs at 3, 10, 30, 50 and 100 kDa. The filtrate was fixed to 2 mL, and then the peptide content was measured by the Folin–Ciocalteu method [28]. The peptide content of the supernatant before ultrafiltration was also determined by this method to obtain the total peptide content.

### 2.4. Raman Spectroscopy

The chemical structures of synthetic HAP, Type I collagen standard, RBC, MRB and RBAS were recorded on a DXR2 Raman spectrometer (Thermo Fisher, Waltham, MA, USA) equipped with a 785 nm laser source, a grating of 400 lines mm^−1^ and an energy of 15.0 mW; the spectra were collected in the wavenumber range of 3100 to 350 cm^−1^, and all the samples were exposed 40 times for 5 s [2]. After scanning, the Raman data were baseline corrected, and phenylalanine (1003 ± 1 cm^−1^) was used as the normalization factor [29]; peak fitting was performed to analyze the content of the secondary structure component of the protein finally, which were all carried out by Origin (2021, Origin-Lab, Northampton, NC, USA).

### 2.5. Particle Size Characteristic Measurements

The Mastersizer 3000 laser diffraction particle size analyzer (measuring range: 10 nm to 3.5 mm; Malvern Panalytical, Nottingham, UK) equipped with a semiautomatic wet sampler (Hydro EV) was used to measure the particle size and distribution according to the method of Ullah et al. [30] with some modifications. Briefly, 3 g of MRB aqueous suspension (5%, *w*/*w*) or RBAS was added to 0.015 g sodium hexametaphosphate (dispersant, 0.5%) and treated with ultrasonic (40 kHz) dispersion at 100 W for 15 min. Finally, the particle size was measured until the shading rate increased to approximately 13% with the sample addition.

A scanning electron microscope (ProX, Phenom, Eindhoven, The Netherlands) was used to observe the microstructure of bone particles, and the parameters were set with a magnification of 7000 and an acceleration voltage of 10.0 kV [2]. The bone suspension was diluted to 50 ppm, dropped onto a 3 mm × 3 mm silicon wafer and dried in natural air. The samples were all sputter-coated with gold before observation.

### 2.6. Stability, Zeta Potential and pH Measurements

The stability of the RBASs was determined by the method of Klavons et al. [21] and Chen et al. [31] with slight changes. Briefly, 10 mL of bone suspension was added to a colorimetric tube, and stratification was observed after standing for 24 h at 4 °C. The height of the lower sediment (H_1_) and the overall height of the suspension (H_2_) were measured. The stability was obtained using Equation (2).

Stability (%) = H_1_/H_2_ × 100% (2)

The RBASs were diluted to 0.1 g/100 g using ultrapure water to determine the zeta potential by a Zetasizer Nano ZS90 analyzer (ZEN3690, Malvern Panalytical, Nottingham, UK), as described by Yin et al. [13]. The pH of the RBASs was assayed with a digital pH meter (S220-K, Mettler Toledo, Switzerland) at room temperature.

### 2.7. Solubility and Chelation Rate Measurements

The protein and calcium solubility of the RBASs were determined according to the method of Chen and Li et al. [3,21] with some modifications. The protein contents in the supernatant (C_1_) and bone suspension (C_2_) were measured by the method in Section 2.3.1, and the protein solubility was calculated using Equation (3).

Protein solubility (%) = C_1_/C_2_ × 100% (3)

The calcium content (C_Ca_) in a certain volume (V_Ca_) of supernatant was measured by the method described in Section 2.3.3, and the calcium solubility was represented as micrograms of soluble calcium per gram of MRB in the bone suspension (M_MRB_), as shown in Equation (4).

Calcium solubility (μg/g) = C_Ca_ × V_Ca_/M_MRB_ (4)

The peptide calcium was separated from the supernatant by absolute ethanol [32]. The supernatant with the same volume (V_Ca_) was added to a centrifuge tube, mixed with absolute ethanol (1:5, *v*/*v*), and then blended thoroughly with a vortex oscillator (MS3, IKA, Staufen, Germany) at 600 rpm for 5 min. Next, the mixture was centrifuged at 8000× *g* for 10 min, and the precipitate was collected. The content of calcium in the precipitate (C_P-Ca_) was determined as described in Section 2.3.3. The chelation rate was calculated using Equation (5).

Chelation rate (%) = C_P-Ca_/C_Ca_ × 100% (5)

### 2.8. Surface Hydrophobicity and Reactive Sulfhydryl Measurements

The method reported by Zhang et al. [33] was used to determine the surface hydrophobicity of RBASs. Briefly, 1 mL of bone suspension was mixed with 0.2 mL of bromophenol blue (BPB, 1 mg/mL), shaken well by a constant temperature shaker at 120 rpm for 10 min, and then centrifuged at 8000× *g* for 15 min. Part of the supernatant was taken and diluted 10 times with PBS (0.02 M). Finally, the amount of bound BPB of diluent was determined by measuring the absorbance at 595 nm (A_control_, A_sample_). Ultrapure water was used as the control instead of bone suspension. The surface hydrophobicity was estimated using Equation (6).

Bound BPB (μg) = 200 μg × (A_control_ − A_sample_)/A_control_ (6)

The reactive sulfhydryl content was determined by using the procedure described by Zhang et al. [33] with slight modifications. Briefly, 1 mL of bone suspension was diluted with 9 mL PBS (0.05 M; containing 8 M urea, 0.6 M NaCl and 0.01 M EDTA-2Na) and vortexed at 600 rpm for 1 min, and then centrifuged at 4000× *g* for 30 min. After that, 3 mL of supernatant was mixed with 0.4 mL of 2-nitrobenzoic acid (0.1%, *w*/*w*), and the absorbance (A_RS_) of the supernatant was measured at 412 nm after incubation at 40 °C for 25 min in the dark. The protein content in the bone suspension (C_2_) was the same at 2.7. The reactive sulfhydryl was calculated using Equation (7).

Sulfhydryl content (nmol/mg) = 10^5^ × A_RS_/(136 × C_2_) (7)

### 2.9. Intermolecular Chemical Force Measurement

The chemical forces of RBAS were measured and calculated according to the method of Zhang et al. [34] with some modifications. Briefly, 1 mL of bone suspension was mixed with 5 mL solution A (0.05 M NaCl), solution B (0.6 M NaCl), solution C (0.6 M NaCl containing 1.5 M urea), solution D (0.6 M NaCl containing 8 M urea) and solution E (0.6 M NaCl containing 8 M urea and 0.05 M β-ME), and homogenized at 7000 rpm for 30 s. Next, the solutions were reacted at 4 °C for 60 min, and then centrifuged at 9600× *g* for 15 min. Finally, the protein content of the supernatant after centrifugation was determined by the Folin–Ciocalteu method [28], and the results are expressed by the mass of dissolved protein in each mL of solution (mg/mL).

### 2.10. Statistical Analysis

All experiments were performed at least in triplicate. The data are presented as the mean ± standard deviation, and the differences between means were analyzed with ANOVA by using SPSS software (Version 19.0, IBM Corp., Armonk, NY, USA). A significant difference was confirmed when the *p* value < 0.05, and an extremely significant difference was established when the *p* value < 0.01 or 0.001. The principal component analysis (PCA), Pearson’s correlation analysis and hierarchical clustering heatmap construction were carried out by the applications in Origin 2021.

## 3. Results and Discussions

### 3.1. Raw Material Characteristics

**Figure 2:**
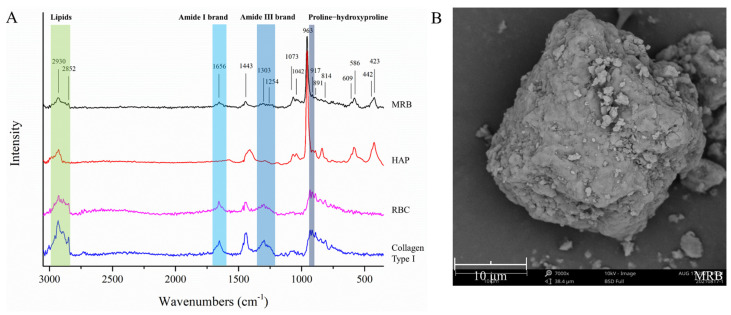
(**A**) Raman spectrogram of MRB: compared with synthetic hydroxyapatite (HAP), RBC and Type I collagen standard (from bovine Achilles tendon). (**B**) Micromorphology of the raw material (MRB).

To preliminarily crush, fresh rabbit bone was prepared into the experimental raw material, which was the MRB (Figure 1). The chemical composition, chemical structure and micromorphology of MRB are shown in Table 1 and Figure 2.

In a previous study [2], the contents of ash, protein, calcium and hydroxyproline (Hyp) in the dry-fat-free basis of fresh rabbit bone were approximately 59.0%, 41.5%, 25.1% and 2.7%, respectively. Since Hyp is a specific amino acid of collagen, 7.1 is often used as a conversion coefficient to calculate the collagen content of terrestrial animals [35]. Therefore, the collagen content in fresh rabbit bone was approximately 19.0%. The protein loss in rabbit bone was substantial and was reduced to approximately 15.9%, as shown in Table 1, which was probably because the high temperature of the pretreatment led to the partial hydrolysis of collagen, and the triple helix decomposed into gelatin [36] and was lost with the subsequent washing process. The analysis of amino acids showed that the content of Hyp in MRB decreased to 1.31%; thus, the content of collagen was approximately 9.3%. The remaining soluble substances of MRB, such as gelatin, were dissolved after washing again with 45 °C hot water, but the content of this soluble component was low (approximately 1.3%). There was no significant difference (*p* > 0.05) between the amino acids composition in MRB before and after hot water washing. Therefore, the gelatin content in MRB was low, and MRB was still difficult to dissolve in water. Due to the concentration effect caused by the loss of protein, the ash and calcium contents in MRB increased to approximately 76.8% and 30.4%.

The Raman spectrum is shown in Figure 2A. To further understand the chemical composition of MRB, synthetic HAP, collagen type I standard and extracted rabbit bone collagen were used as the control groups. Collagen mainly exists in the form of fibrous type I collagen in the bones and tendons of animals [37]. The spectrum was similar between RBC and the type I collagen standard, indicating that the collagen in rabbit bone was mainly type I collagen. The lipid region, amide I and III region and proline-hydroxyproline (Pro-Hyp) region are the main regions for conformational changes of collagen structure [38]; the 1443 and 891 cm^−1^ regions are two Raman bands in the vibrational region of carbonate ions [39], and these bands were completely present in the profiles of RBC and the type I collagen standard. In the Raman spectrum of HAP, the occurrence positions of a strong peak, moderate intensity peaks and lower intensity peaks are similar to the structural distribution of synthetic and natural HAP previously studied [40,41]. The Raman spectrum waveform of MRB basically corresponded to the characteristic peaks of HAP and RBC. Therefore, MRB was mainly composed of HAP and type I collagen. Certainly, the large loss of collagen led to the obvious reduction in the characteristic peaks of the amide and Pro-Hyp regions. It is generally believed that the closer the intensity ratio of the amide III band (1300 cm^−1^, measured in the experiment) and the peak near 1450 cm^−1^ (I_1300 cm−1_/I_1450 cm−1_) is to 1, the more complete the triple helix structure of collagen [42]. The ratios were approximately 0.85 in RBC and approximately 0.78 in MRB, indicating that the collagen remaining in the MRB retained the triple helix structure, which was consistent with the results of the chemical analysis.

In Figure 2B, it could be seen that the MRB particles were irregular polygons, presenting a dense structure formed by the deposition of mineral salts such as HAP in the collagen network [4]. Some small particles adhered to the surface, which might be caused by van der Waals forces [12,43]. The average particle size of the MRB was approximately 32.9 μm (detected by Malvern 3000), which was consistent with that observed by SEM. However, the particle size was still large for the HPH equipment. The MRB needed to be ball milled with ultrapure water to prepare a bone suspension with a particle size of 10.40 ± 0.01 μm, which was the control group.

Overall, MRB was a raw material rich in calcium and protein, and collagen was still the main protein, accounting for nearly 58.6% of the total protein (9.31/15.90 × 100%).

### 3.2. Effect of HPH Cycles on the Quality Properties of RBAS

**Figure 3:**
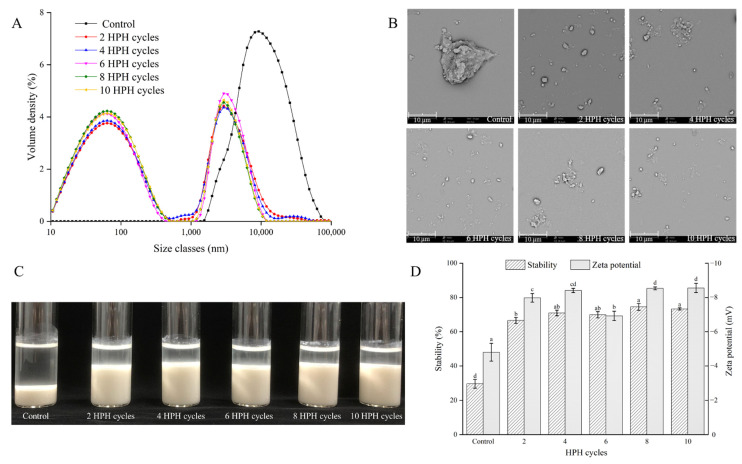
Effects of HPH on the particle size distribution (**A**) and microstructure (**B**) of RBAS. (**C**) Photo of RBAS treated with different HPH cycles after standing at 4 °C for 24 h. (**D**) Stability (standing at 4 °C for 24 h) and zeta potential of RBAS. In figure, different lowercases in each group showed significant differences (*p* < 0.05).

Fine, smooth taste and good stability are two very important quality characteristics of an oral suspension. Therefore, the particle size characteristics and stability of the RBASs were selected as the main quality indicators. The particle size distribution and microscopic morphology of the RBASs after different numbers of HPH cycles are shown in Figure 3 and Table 2.

In Figure 3A, the particles of the control group were all distributed in the micron-scale range, presenting a single peak. After two cycles, the peak shifted significantly to the nanoscale, showing double peaks, and most of the particles were distributed at the nanoscale. With the increase in HPH cycles, the peak volume density of the nanoscale increased continuously and reached the highest value at eight cycles but then decreased at ten cycles. Table 2 also shows that as the number of cycles increased to eight, the Dx (50) of bone particles decreased significantly (*p* < 0.05), and the specific surface area increased continuously (*p* > 0.05). The proportion of particles distributed at the nanoscale also reached approximately 65.7%, which was the particle size limit. In the research of Chen and Yin et al. [13,21], the particle size of large particles in the dispersion system was found to be effectively reduced by HPH treatment, which indicated that HPH was a means to significantly reduce the particle size of the dispersion system. However, the particle characteristics decreased when there were too many at ten cycles. The small-size effect caused the nanoscale bone particles to have higher interfacial energy, which would lead to particle aggregation [10]. With nanogrinding, the particles establish a balance between agglomeration and disaggregation until reaching the limit particle size [3]. As shown in Figure 3B, the particles agglomerated with decreasing particle size.

The stability changes of the RBAS after standing at 4 °C for 24 h are shown in Figure 3C,D. After HPH treatment, the RBAS was uniform milky white (Figure 1). However, the RBAS became layered after standing for hours, as shown in Figure 3C, which was the precipitation phenomenon caused by the different densities of particles and media [44]. Delamination was significantly improved by HPH treatment, and the transparent water layer decreased with increasing cycles. Similar situations also occurred during the processing of whey protein, inulin and fruit juice [20,44,45]. HPH treatment could significantly improve the uniformity and stability of the liquid and reduce precipitation. However, this influence was not infinite, and the transparent water layer was no longer reduced at eight HPH cycles. As shown in Figure 3D, the stability of the bone suspension was no longer significantly improved (*p* > 0.05) after eight cycles, which might be related to the presence of some micron-sized particles in the suspension, and the particle size reached a limit at this time. The zeta potential results also confirmed this phenomenon. Zeta potential is an important indicator of the stability in a dispersion system and is related to the charged residues on the surfaces of the particles in suspension [46]. As shown in Figure 3D, the absolute value no longer increased (*p* > 0.5) and reached the limit value of approximately 8.5 at eight HPH cycles. Interestingly, the absolute value decreased significantly (*p* < 0.5) at six HPH cycles, which might be related to the agglomeration of particles. As shown in Figure 3C, it was also observed that the transparent water layer did not decrease significantly after six HPH cycles. However, the absolute value increased, and the delamination decreased when the cycles reached eight, which might be due to the re-dispersion of agglomerated particles and the exposure of surface charges. Therefore, HPH was an effective method to improve the storage stability of RBASs, and the effect was the best at eight HPH cycles.

### 3.3. Effect of HPH Cycle on the Dissolution Characteristics of RBAS

**Figure 4:**
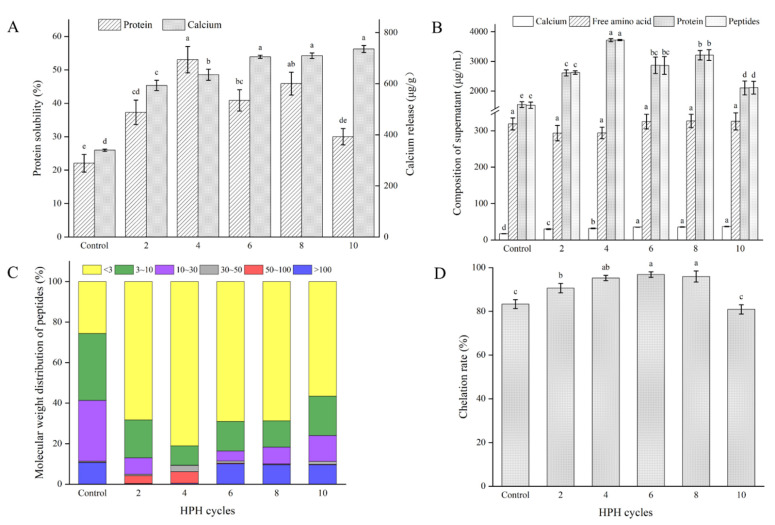
Solubility (**A**) and content (**B**) of the main components of RBAS after different HPH cycles. Molecular weight distributions of peptides (**C**) and chelation rate of peptide calcium (**D**) in RBAS treated with different HPH cycles.In figure, different lowercases in each group showed significant differences (*p* < 0.05).

Previous studies have confirmed that HPH has solubilizing effects on protein and calcium [14,20,21]. Overall, HPH significantly increased the solubility of protein and calcium in RBAS (*p* < 0.05), as shown in Figure 4A. The protein solubility in the control was approximately 22.1%. When the number of HPH cycles reached four, the protein solubility increased to approximately 53.1% but decreased at six and ten cycles. It was similar to the changing trend of stability, which might be due to the “overprocessing” effect. After protein reaggregation, water-insoluble aggregates were produced [21]. The generation of aggregates did not affect the particle size until ten HPH cycles, as shown in Table 2, which might be due to the large particle size and number of aggregates. The solubility of proteins is related to protein structure, interactions between protein molecules and water and pH, among others. [46,47,48]. However, the pH of the bone suspension did not change significantly and remained neutral, as shown in Table 2. There was no change in the pH when Chen et al. [21] prepared a chicken MP aqueous suspension with HPH. Before HPH treatment, the solubility of calcium was approximately 339.1 μg/g. With the increases in HPH cycles, the solubility of calcium continuously increased (*p* < 0.05) until approximately 705.0 μg/g at six cycles, the improvement of which might be mainly due to the increase in the specific surface area of the HAP particles [10]. When the bone particles reached the particle size limit, the release of calcium ions also tended to become stable. Although there were obvious, large aggregate proteins produced at ten cycles, the content of calcium ions was not affected, which indicated that they might have been released from HAP.

Compared with the control, the contents of calcium, protein and peptides in the supernatants after HPH treatment increased (*p* < 0.05), especially the contents of protein and peptides, as shown in Figure 4B. It is worth mentioning that there was no significant difference (*p* > 0.05) between the contents of dissolved protein and total peptides, so it could be inferred that the dissolved proteins in the supernatant were mainly peptides. Li et al. [3] also found that high-energy wet ball milling led to the degradation of some fish bone collagen into peptides. However, unlike her research, the content of free amino acids and the pH in RBAS were not significantly increased (Table 2). Therefore, HPH and high-energy wet ball milling had different effects on animal bone.

The molecular weight distribution of the peptides in RBAS is shown in Figure 4C and Table 3. It is meaningful that the content of peptides less than 3 kDa was increased significantly by HPH treatment, and the effect was best at four HPH cycles, with the content increased from approximately 395.6 μg/mL to 3008.7 μg/mL, accounting for approximately 81.0% of the total peptides. The analysis of amino acids in the supernatant is shown in Table 3. Gly, Pro and Hyp were the main amino acids, as Gly-Pro-Hyp is the characteristic sequence of type I collagen, indicating that there were many collagen peptides in the supernatant. Currently, oral collagen peptides with a molecular weight of less than 3 kDa are popular. A large number of studies have shown that small molecular collagen peptides can be better absorbed, alleviate arthritis and effectively improve bone calcium deficiency, and have the effects of antioxidation, antiaging, whitening and relieving skin dryness [49,50,51,52,53,54]. However, the contents of the total peptides and small-molecule peptides in the supernatant decreased, while the content of the peptides greater than 100 kDa increased at six HPH cycles, which further explained the aggregation of the protein. The aggregates of macromolecules were separated from the solution, which not only affected the particle size and stability of the bone suspension but also led to a significant decrease in the content of soluble peptides at ten HPH cycles.

Figure 4D shows the chelation of calcium ions and peptides in the supernatant. In the control, approximately 83.3% of calcium was chelated with peptides, and the chelation rate was as high as 96.9% at six HPH cycles. It could be seen that the dissolved calcium mainly existed in the form of chelation with peptides. Soluble peptides chelating calcium have the characteristics of high stability, a high calcium absorption rate and high bioavailability [55]. However, the chelation rate decreased to approximately 80.9% (*p* < 0.05) at ten HPH cycles. There is a strong relationship between the chelation ratio and the peptide–calcium coordination ratio [56]. Although HPH treatment resulted in more calcium ions being released from rabbit bone, the generation of protein aggregates caused a decrease in peptide content at ten HPH cycles, which might lead to a significant decrease in the chelation rate.

In summary, on the one hand, HPH promoted the dissolution of protein in RBASs, degraded a portion of the collagen into collagen peptides, and mainly produced small-molecule peptides (<3 kDa); on the other hand, HPH promoted the release of calcium ions from rabbit bone, and most calcium ions chelated with peptides to generate peptide calcium. However, too many cycles of HPH will reduce the protein solubility and the chelation rate.

### 3.4. Changes in Protein Conformation in RBAS

**Figure 5:**
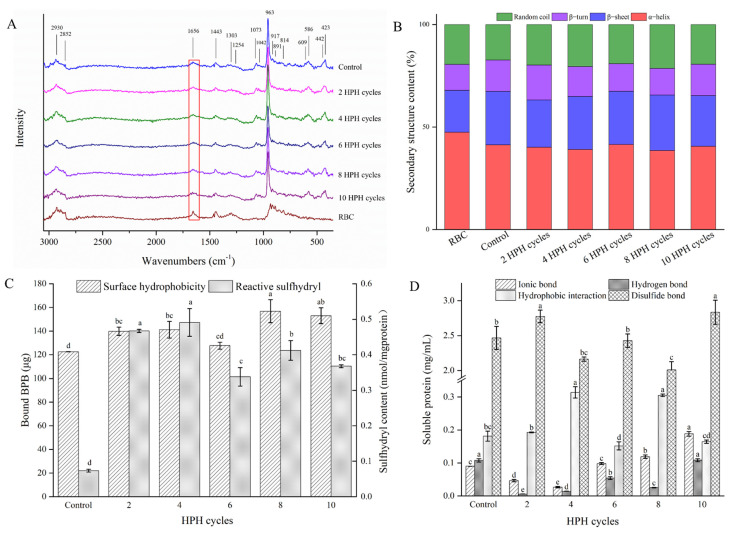
Chemical structures and intermolecular chemical forces of RBASs under different HPH cycles. (**A**) Raman spectra of RBC and RBASs. (**B**) Secondary structures of proteins in RBAS. Effect of HPH on the surface hydrophobicity, reactive sulfhydryl content (**C**) and intermolecular chemical force (**D**) of proteins in RBASs. In figure, different lowercases in each group showed significant differences (*p* < 0.05).

The strong mechanical effect produced by HPH affected the protein solubility of chicken breast MP and soybean protein isolate by dissociating or fragmenting the highly ordered structure of macromolecules [21,57]. The chemical structure of the RBASs changed, especially in the amide region of the Raman spectrum, as shown in Figure 5A.

The signals of the amide I region are usually used to analyze the relative content of the secondary structure [58,59]. The order of each secondary structure appearing in the amide I region of the Raman is α-helix 1645–1660 cm^−1^, random coil 1660–1665 cm^−1^, β-sheet 1665–1680 cm^−1^ and β-turn 1680–1690 cm^−1^ [60,61,62]. On the whole, α-helix decreased significantly, β-sheet showed a decreasing trend and random coil increased significantly, whereas β-turn first increased, then decreased and increased again, as shown in Figure 5B. Consequently, α-helix and β-sheet decreased and added to the random coil and β-turn structure transformation. The α-helix and β-sheet structures are stabilized by hydrogen bonds within and between the peptide chains, respectively [63], and the random coil structure comes from the unfolding of higher-order structures of protein and is able to reflect the flexibility degree of the peptide chains, while the β-turn structure is derived from highly ordered protein structures [64,65]. This illustrated that HPH might disrupt the hydrogen bonds of peptide chains and the intact and ordered structure of bone protein, leading to protein denaturation, unfolding and accelerating the movement of protein molecules, thus promoting the dissolution of rabbit bone protein in water. A similar phenomenon was found in the MP of chicken breast; HPH treatment destroyed the α-helix structure, led to the denaturation and unfolding of protein and improved the solubility of MP [21]. In addition, the release of calcium ions also likely directly disrupted the α-helix structure [66], which would further promote the dissolution of protein. The α-helix content increased and was accompanied by a decrease in the random coil at six HPH cycles, at which point the protein solubility decreased. When the number of HPH cycles reached ten, both the α-helix and β-turn structures increased, the random coil and β-sheet structures decreased simultaneously and the solubility of the protein decreased considerably. These results suggested that the protein aggregation induced by HPH overtreatment might be due to the rearrangement of the disordered structure and the increase in the ordered structure of the protein, resulting in a decrease in protein solubility. Gao et al. [67] found the solubility of cowhide gelatin induced by high hydrostatic pressure first increased and then decreased with the increase in pressure (200–400 MPa); at the same time, the α-helix structure first decreased and then increased. The opposite was true for the random coil structure, which converts, causing the structure of the gel to be more compact and orderly. Guo et al. [68] also found a similar situation when using HPH (30–120 MPa) to treat thermally soluble aggregated kidney bean proteins; the HPH at a lower pressure could destroy the protein aggregates mainly manifested as the increase in intermolecular β-sheet and random coil structures. However, the intermolecular β-sheet and random coil structures decreased while the α-helix and β-turn structures increased with the excessive pressure.

The extent of the denatured unfolding of protein could be reflected by surface hydrophobicity and the content of reactive sulfhydryl. Figure 5C shows that both surface hydrophobicity and reactive sulfhydryl content of RBASs were significantly increased overall after HPH treatment (*p* < 0.05), which was similar to the study of Chen et al. [21]. The intense shear stress and cavitation produced by HPH led to the dispersion of aggregated particles, depolymerization or rearrangement in the protein system, which caused the denaturation and structural unfolding of the protein and the exposure of amino acid residues buried in the protein’s interior [69]. From the amino acid analysis of MRB (Table 1), the sulfur-containing amino acids in rabbit bone were determined to be mainly Met and Cys, and the hydrophobic amino acids were mainly Leu, Val and Ile. Therefore, these amino acid residues might be exposed by HPH treatment. The hydrophobic and sulfhydryl contents of the RBASs were reduced significantly (*p* < 0.05) at six and ten HPH cycles, which was consistent with the change in the random coil content. This was probably due to the reaggregation of the protein, resulting in the exposed residues being buried inside the protein again [70].

The tertiary structure of the protein is mainly stabilized by secondary and disulfide bonds. The changes in the chemical forces among the proteins in RBASs are shown in Figure 5D. With the increase in HPH cycles, the content of ionic bonds decreased first and then increased, indicating that HPH could decrease the electrostatic effect between molecules; however, the intermolecular electrostatic effect was enhanced, and the protein aggregated after overtreatment. In addition, calcium ions might form calcium bridges between proteins with a net negative charge and increased electrostatic interactions [71]. The hydrogen bond content generally decreased because these bonds are closely related to the stability of the secondary and tertiary structures of proteins but increased at six and ten HPH cycles, and the changing trend was similar to that of the α-helix structure. The hydrophobic interactions were highest at four and eight HPH cycles, which is consistent with the trend of surface hydrophobicity. This is probably because the protein structure changed and the amino acid hydrophobic residues were exposed, which led to the increase in hydrophobic interactions [72,73]. The content of disulfide bonds improved at two HPH cycles, which could be due to the sulfur-containing amino acids (Cys) becoming exposed and disulfide bonds being created at the same time [74], while the disulfide bonds were destroyed and the protein degraded with the increase in HPH cycles. However, the disulfide bonds increased again at six and ten cycles, which might be caused by protein aggregation. In summary, appropriate amounts of HPH cycling could disrupt the hydrogen bonds, ionic bonds and disulfide bonds of rabbit bone protein, improve hydrophobic interactions, and then cause the protein to degrade, disperse and dissolve in water, but overtreatment might result in the reorganization of amino acid residues on peptide chains through these bonds.

### 3.5. Analysis of Correlation

**Figure 6:**
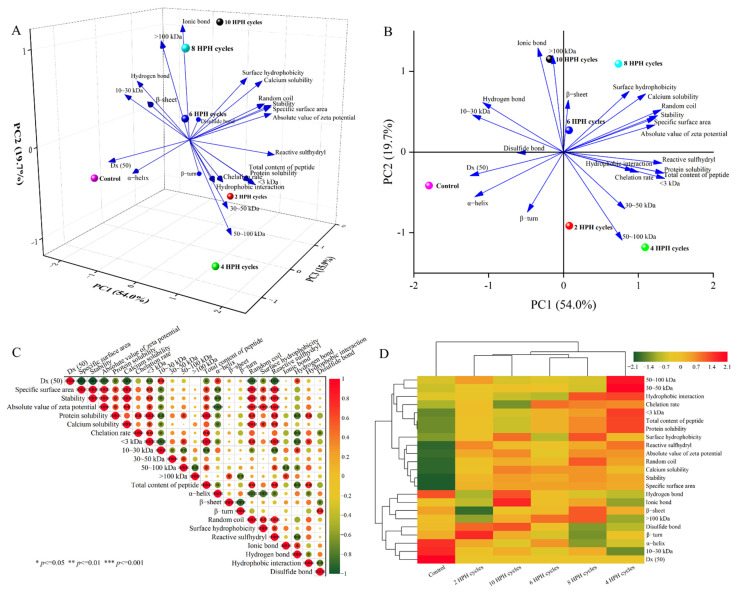
Three−dimensional (**A**) and two−dimensional (**B**) biplots (score and loading) from principal component analysis (PCA) of RBASs show the relationship between quality properties, dissolution characteristics, protein conformation and different HPH treated methods. Correlation heatmap (**C**) among quality properties, dissolution characteristics and protein conformation of RBASs. The color gradation and circle size correspond to the Pearson’s correlation coefficient (r), while the pattern with the “*, **, ***” mark represents a significant (*p* ≤ 0.05) or extremely significant difference (*p* ≤ 0.01 or 0.001). Clustering heatmap (**D**) of quality properties, dissolution characteristics and protein conformation of RBASs. Rows and columns are grouped by correlation distance and average linkage, respectively. The color gradation represents the Z-scores of corresponding parameters.

The PCA model, Pearson’s correlation analysis and clustering heatmap of significantly changed physicochemical indicators shown in Figure 6 further analyzed the relationship between the quality, solubility and protein conformation of RBASs. Figure 6A is a 3D biplot of the variable loading and sample score, in which 89.5% of the total variance could be explained by the first three principal components. Therefore, there was a strong correlation between these physicochemical indices of RBASs. For better observation, the 2D biplot of PC1 and PC2 is shown in Figure 6B. PC1 (54.0%) was mainly highly correlated with small-molecule peptide (<100 kDa) content, total peptide content, the solubility of protein and calcium, reactive sulfhydryl, surface hydrophobicity, random coil, α-helix, stability, Dx (50), specific surface area, the absolute value of zeta potential, hydrogen bond, hydrophobic interaction and chelation rate, which could reflect the quality characteristics, dissolution characteristics and major conformational changes of RBASs. Among them, Dx (50), the peptide content at 10–30 kDa, α-helix and hydrogen bonds were negatively correlated with PC1, and the other indices were positively correlated with PC1. PC2 (19.7%) was mainly positively correlated with ionic bonds and peptide content greater than 100 kDa, which might mainly reflect the production of protein aggregates. PC3 (15.9%) was mainly positively correlated with disulfide bonds and β-turn and negatively correlated with the β-sheet, which might mainly represent some protein conformations that had less influence on the overall characteristics of RBASs. In addition, the points of the sample were far apart in the 3D PCA model, which illustrated the large intersample variation. The loading of Dx (50), α-helix and β-turn were closest to the control group, which indicated that these indicators had the highest impact on it. At HPH cycles two and four, the sample points were close to the loading of the small-molecule peptide content, total peptide content, solubility of protein, chelation rate and hydrophobic interaction, while the sample point was close to those of β-sheet and the disulfide bond at six HPH cycles. The sample was favorably affected by surface hydrophobicity, the solubility of calcium, random coil, stability, specific surface area and the absolute of zeta potential at eight HPH cycles. The loading of ionic bonds and a peptide content greater than 100 kDa affected the sample the most at ten HPH cycles. These results demonstrated once again that HPH could reduce the particle size and disrupt the protein structure in RBASs significantly, leading to protein unfolding and degradation, and improving the solubility and stability of rabbit bone particles. However, excessive treatment with HPH might cause protein reaggregation due to intermolecular forces.

The correlation between indices could be explained by Pearson’s correlation matrix, as shown in Figure 6C. There was a strong correlation between the particle size, stability and solubility of RBASs. The indicators of the protein conformation, such as α-helix, random coil, surface hydrophobicity, reactive sulfhydryl, ionic bond, hydrogen bond and hydrophobic interaction, had strong correlations with not only the quality properties of RBASs but also the protein solubility. This indicated that the improvement of the quality properties of RBASs could be attributed to the improvement of solubility, which was strongly associated with the change in protein conformation, while the improvement of calcium solubility was associated with the improvement of the quality properties of RBASs. Therefore, it could be deduced that the changes in protein conformation affected the solubility of the protein in water, thus improving the quality of RBASs, which in turn led to an increase in calcium solubility.

As shown in the clustering heatmap in Figure 6D, all the physicochemical characteristics were mainly dispersed into two clusters with a Euclidean distance <3.1. These two groups were mainly characterized by whether they were positively or negatively correlated with the overall characteristics of RBASs, which was similarly observed in the studies by Zhang et al. [10] and Zhuang et al. [75]. The samples were divided into four groups with a Euclidean distance <5.1. The comprehensive characteristics of RBASs were sorted as follows: Group 1 (control) < Group 2 (two and ten HPH cycles) < Group 3 (six and eight HPH cycles) < Group 4 (four HPH cycles). Certainly, this type of ranking was based on the equal importance of each index, and no corresponding weight was given to each characteristic. These results proved once again that HPH had a significant effect on improving the quality of RBASs. However, when the number of HPH cycles was too high, the overall quality of the RBASs decreased.

## 4. Conclusions

The strongly mechanical action induced by HPH mainly broke the hydrogen bonds and strengthened the hydrophobic interactions among the protein molecules and transformed the partial α-helix structure of the protein into a random coil structure. These resulted in the denaturation, unfolding and degradation of protein and the formation of small-molecule soluble peptides, which further enhanced the solubility of rabbit bone protein. Meanwhile, the increase in the surface charge of protein molecules could improve the stability of RBASs. The embedded HAP was released because of the partial dissolution of protein (e.g., collagen), and the size of the particles was further reduced due to the shearing effect of HPH, which resulted in the generation of a large number of bone nanoparticles. Therefore, the specific surface area of the particles increased, resulting in the release of calcium ions from HAP into water dispersion. The corresponding calcium ions could chelate with peptides to generate peptide calcium, which ultimately improved the solubility of calcium in RBASs. However, excessive HPH cycles could lead to the formation of water-insoluble macromolecular aggregates. The overall quality of the bone aqueous suspension was best at the condition of four HPH cycles, and better stability could be achieved at eight HPH cycles. This study not only contributes to our understanding of the mechanism of nanoscale bone preparation by HPH technology but also helps to promote the application of nanoscale animal bone in foods.

## Figures and Tables

**Table 1:** Chemical composition of micron-scale rabbit bone (MRB).

Items	MRB	MRB Eluted by Hot Water (45 °C)
Proximate (%)		
Fat	0.81 ± 0.16	/
Moisture	4.98 ± 0.03	/
Total Protein	15.90 ± 0.20	/
Ash	76.80 ± 0.07	/
Soluble component	1.33 ± 0.18	/
Minerals		
Calcium (mg/g)	303.70 ± 1.27	/
P (mg/g)	172.45 ± 2.90	/
Magnesium (mg/g)	5.07 ± 0.19	/
Zinc (mg/kg)	243.10 ± 3.39	/
Amino acids (%)		
Gly	2.65 ± 0.10	2.58 ± 0.11
Pro	1.53 ± 0.08	1.54 ± 0.05
Hyp	1.31 ± 0.03	1.26 ± 0.05
Glu	2.04 ± 0.07	2.05 ± 0.09
Ala	1.26 ± 0.04	1.25 ± 0.06
Arg	1.14 ± 0.03	1.13 ± 0.11
Asp	0.70 ± 0.03	0.72 ± 0.02
Lys	0.79 ± 0.04	0.79 ± 0.02
Leu	0.80 ± 0.05	0.85 ± 0.04
Ser	0.60 ± 0.02	0.60 ± 0.03
Phe	0.46 ± 0.02	0.47 ± 0.02
Val	0.59 ± 0.03	0.61 ± 0.03
Thr	0.51 ± 0.02	0.52 ± 0.03
Ile	0.32 ± 0.02	0.33 ± 0.02
Tyr	0.42 ± 0.07	0.47 ± 0.04
His	0.20 ± 0.01	0.21 ± 0.01
Met	0.03 ± 0.00	0.03 ± 0.01
Cys	0.04 ± 0.01	0.04 ± 0.00
Tau	0.02 ± 0.01	0.01 ± 0.00
Total EAA	3.51 ± 0.02	3.53 ± 0.03
Total NEAA	11.92 ± 0.04	11.86 ± 0.06

The “/” indicates that the indicator was not measured.

**Table 2:** Particle size characteristics and pH of rabbit bone aqueous suspensions (RBAS).

HPH Cycles	Dx (50) (nm)	Specific Surface Area (m^2^/kg)	Proportion within 100 nm (%)	Proportion within 1000 nm (%)	pH
Control	10,400 ± 0 ^a^	721 ± 1 ^f^	0.00 ± 0.00 ^e^	0.00 ± 0.00 ^f^	6.97 ± 0.01 ^a^
2	165 ± 2 ^b^	73,653 ± 278 ^e^	44.45 ± 0.17 ^d^	59.28 ± 0.23 ^e^	6.81 ± 0.01 ^a^
4	152 ± 1 ^c^	75,670 ± 211 ^d^	45.62 ± 0.13 ^c^	61.33 ± 0.17 ^d^	7.04 ± 0.01 ^a^
6	127 ± 2 ^d^	81,190 ± 316 ^b^	48.93 ± 0.20 ^b^	62.37 ± 0.40 ^c^	6.81 ± 0.01 ^a^
8	121 ± 1 ^e^	82,670 ± 17 ^a^	49.86 ± 0.01 ^a^	65.69 ± 0.07 ^a^	7.00 ± 0.01 ^a^
10	128 ± 1 ^d^	80,803 ± 21 ^c^	48.75 ± 0.02 ^b^	64.80 ± 0.07 ^b^	7.00 ± 0.01 ^a^

Different lowercase superscripts in the same column indicate significant differences (*p* < 0.05).

**Table 3:** Peptide contents with different molecular weights and amino acid compositions in the supernatants of RBAS.

Items	Control	Two HPH Cycles	Four HPH Cycles	Six HPH Cycles	Eight HPH Cycles	Ten HPH Cycles
Molecular Weight (kDa)	Peptides Contents (μg/mL)					
<3	395.60 ± 51.44 ^e^	1782.92 ± 166.58 ^c^	3008.66 ± 202.41 ^a^	1975.22 ± 150.95 ^bc^	2209.38 ± 158.69 ^b^	1186.53 ± 92.35 ^d^
3~10	511.01 ± 58.00	487.92 ± 62.76	360.82 ± 77.11	416.76 ± 35.09	415.52 ± 32.74	408.22 ± 30.05
10~30	461.49 ± 51.90 ^a^	210.89 ± 13.64 ^bc^	4.25 ± 1.08 ^d^	144.03 ± 76.79 ^c^	266.03 ± 32.43 ^b^	267.94 ± 27.90 ^b^
30~50	8.04 ± 2.47 ^d^	18.58 ± 1.53 ^c^	36.24 ± 2.79 ^a^	28.70 ± 1.88 ^b^	9.91 ± 1.38 ^d^	31.32 ± 4.56 ^ab^
50~100	3.01 ± 1.66 ^c^	102.18 ± 3.21 ^b^	213.19 ± 9.98 ^a^	9.43 ± 3.01 ^c^	4.36 ± 1.57 ^c^	4.02 ± 1.27 ^c^
>100	165.20 ± 21.96 ^c^	7.83 ± 3.59 ^d^	16.72 ± 3.93 ^d^	286.95 ± 4.41 ^a^	307.82 ± 7.79 ^a^	200.95 ± 12.49 ^b^
Total	1544.34 ± 183.10 ^e^	2610.32 ± 258.33 ^cd^	3713.88 ± 278.78 ^a^	2861.09 ± 222.52 ^bc^	3213.02 ± 237.6 ^ab^	2098.97 ± 171.62 ^de^
	Amino Acids Contents (μg/mL)					
Gly	366.58 ± 18.82 ^f^	613.53 ± 17.54 ^d^	821.36 ± 9.25 ^a^	693.50 ± 17.35 ^c^	744.79 ± 25.81 ^b^	512.62 ± 22.47 ^e^
Pro	188.09 ± 3.6 ^e^	335.17 ± 20.42 ^c^	463.00 ± 24.35 ^a^	368.99 ± 6.34 ^bc^	394.93 ± 12.48 ^b^	269.18 ± 7.98 ^d^
Hyp	174.93 ± 10.09 ^e^	314.40 ± 4.48 ^c^	401.70 ± 7.24 ^a^	335.05 ± 21.04 ^bc^	359.51 ± 7.94 ^b^	261.86 ± 0.87 ^d^
Glu	181.87 ± 4.86 ^e^	294.47 ± 14.97 ^c^	406.70 ± 13.18 ^a^	320.94 ± 16.87 ^c^	361.77 ± 13.68 ^b^	236.41 ± 8.35 ^d^
Ala	150.58 ± 4.8 ^f^	228.54 ± 10.53 ^d^	310.02 ± 2.9 ^a^	252.36 ± 13.4 ^c^	284.33 ± 7.67 ^b^	195.79 ± 10.81 ^e^
Arg	129.96 ± 5.23 ^d^	160.48 ± 3.48 ^c^	244.46 ± 2.6 ^a^	149.49 ± 6.6 ^c^	193.84 ± 7.71 ^b^	148.94 ± 1.93 ^c^
Asp	60.98 ± 1.32 ^e^	105.58 ± 1.01 ^c^	146.68 ± 6.11 ^a^	112.16 ± 5.75 ^c^	123.97 ± 6.53 ^b^	85.30 ± 2.69 ^d^
Lys	62.58 ± 3.33 ^f^	106.21 ± 0.52 ^d^	177.79 ± 1.86 ^a^	117.34 ± 4 ^c^	150.21 ± 5.02 ^b^	81.24 ± 1.82 ^e^
Leu	54.04 ± 1.23 ^f^	97.10 ± 1.42 ^d^	151.49 ± 2.06 ^a^	108.95 ± 2.46 ^c^	131.86 ± 3.74 ^b^	70.95 ± 3.52 ^e^
Ser	57.24 ± 1.67 ^f^	96.88 ± 1.08 ^d^	135.57 ± 6.01 ^a^	104.66 ± 0.61 ^c^	115.28 ± 4.37 ^b^	75.55 ± 0.24 ^e^
Phe	28.09 ± 0.89 ^f^	52.36 ± 0.67 ^d^	81.49 ± 3.87 ^a^	58.05 ± 0.41 ^c^	69.39 ± 1.66 ^b^	39.00 ± 0.53 ^e^
Val	51.56 ± 0.95 ^e^	86.07 ± 5.3 ^c^	132.23 ± 7.89 ^a^	96.27 ± 6.06 ^c^	114.15 ± 3.34 ^b^	69.05 ± 0.56 ^d^
Thr	41.96 ± 0.7 ^e^	72.50 ± 0.06 ^c^	102.23 ± 0.35 ^a^	76.44 ± 2.13 ^c^	86.46 ± 4.33 ^b^	56.87 ± 1.98 ^d^
Ile	22.58 ± 0.84 ^f^	42.82 ± 1.8 ^d^	63.34 ± 2.18 ^a^	48.40 ± 1.33 ^c^	57.32 ± 0.45 ^b^	33.04 ± 0.38 ^e^
Tyr	8.18 ± 0.35 ^d^	14.84 ± 0.57 ^c^	28.71 ± 0.24 ^a^	15.36 ± 0.88 ^c^	19.64 ± 0.45 ^b^	8.12 ± 0.16 ^d^
His	10.84 ± 0.56 ^d^	20.78 ± 0.07 ^c^	32.97 ± 1.41 ^a^	19.29 ± 0.96 ^c^	24.63 ± 0.73 ^b^	1.62 ± 0.1 ^e^
Met	4.80 ± 0.28 ^e^	15.90 ± 0.2 ^d^	30.19 ± 1.75 ^a^	20.72 ± 0.02 ^c^	22.86 ± 0.84 ^b^	5.42 ± 0.26 ^e^
Tau	3.91 ± 0.08 ^b^	3.60 ± 0.05 ^c^	4.82 ± 0.17 ^a^	2.32 ± 0.1 ^d^	2.25 ± 0.11 ^d^	0.54 ± 0.01 ^e^
Total	1624.89 ± 57.4 ^e^	2682.86 ± 48.11 ^c^	3768.08 ± 232.16 ^a^	2929.75 ± 176.7 ^c^	3303.40 ± 141.45 ^b^	2197.54 ± 29.06 ^d^

Different lowercase superscripts in the same row indicate significant differences (*p* < 0.05).

## Data Availability

Data are contained within the article.

## References

[1] Field R.A., Riley M.L., Mello F.C., Corbridge M.H., Kotula A.W. (1974). Bone composition in cattle, pigs, sheep and poultry. J. Anim. Sci..

[2] Li X., He Z., Xu J., Zhang L., Liang Y., Yang S., Wang Z., Zhang D., Gao F., Li H. (2021). Effect of nanoprocessing on the physicochemical properties of bovine, porcine, chicken, and rabbit bone powders. Food Sci. Nutr..

[3] Li J., Yin T., Xiong S., Huang Q., You J., Hu Y., Liu R., Li Y. (2020). Mechanism on releasing and solubilizing of fish bone calcium during nano-milling. J. Food Process Eng..

[4] Wang X.M., Cui F.Z., Ge J., Wang Y. (2004). Hierarchical structural comparisons of bones from wild-type and liliput(dtc232) gene-mutated Zebrafish. J. Struct. Biol..

[5] Moss M.L. (1961). Studies of the acellular bone of teleost fish. Acta Anat.

[6] Olszta M.J., Cheng X.G., Jee S.S., Kumar R., Kim Y.Y., Kaufman M.J., Douglas E.P., Gower L.B. (2007). Bone structure and formation: A new perspective. Mat. Sci. Eng. R..

[7] Chen Q., Hou H., Wang S., Zhao X., Li B. (2017). Effects of early enteral nutrition supplemented with collagen peptides on post-burn inflammatory responses in a mouse model. Food Funct..

[8] Koizumi S., Inoue N., Shimizu M., Kwon C., Kim H., Park K.S. (2018). Effects of dietary supplementation with fish scales-derived collagen peptides on skin parameters and condition: A randomized, placebo-controlled, double-blind study. Int. J. Pept. Res. Ther..

[9] Leon-Lopez A., Fuentes-Jimenez L., Hernandez-Fuentes A.D., Campos-Montiel R.G., Aguirre-Alvarez G. (2019). Hydrolysed collagen from sheepskins as a source of functional peptides with antioxidant activity. Int. J. Mol. Sci..

[10] Zhang J., Zhu L., Li H., Tang H., Yang H., Zhao K., Kong F., Yin T., Yao Q., Chen L. (2022). Effects of micro-/nano-scaled chicken bones on heat-induced gel properties of low-salt pork batter: Physicochemical characteristics, water distribution, texture, and microstructure. Food Chem..

[11] Li S.B., He Z.F., Li H.J. (2018). Effect of nano-scaled rabbit bone powder on physicochemical properties of rabbit meat batter. J. Sci. Food Agric..

[12] Yin T., Du H., Zhang J., Xiong S. (2016). Preparation and characterization of ultrafine fish bone powder. J. Aquat. Food Prod. T..

[13] Yin T., Park J.W., Xiong S. (2015). Physicochemical properties of nano fish bone prepared by wet media milling. LWT-Food Sci. Technol..

[14] Wang Y., Feng T., Xia Q., Zhou C.Y., Cao J.X. (2022). The Influence of comminuting methods on the structure, morphology, and calcium release of chicken bones. Front. Nutr..

[15] Yin T., Park J.W., Xiong S. (2017). Effects of micron fish bone with different particle size on the properties of silver carp (hypophthalmichthys molitrix) Surimi gels. J. Food Qual..

[16] Wan J., Liu C.M., Liu W., Tu Z.C., Wu W., Tan H.Z. (2015). Optimization of instant edible films based on dietary fiber processed with dynamic high pressure microfluidization for barrier properties and water solubility. LWT-Food Sci. Technol..

[17] Kasemwong K., Ruktanonchai U.R., Srinuanchai W., Itthisoponkul T., Sriroth K. (2011). Effect of high-pressure microfluidization on the structure of cassava starch granule. Starch-Starke.

[18] Dumay E., Chevalier-Lucia D., Picart-Palmade L., Benzaria A., Gracia-Julia A., Blayo C. (2013). Technological aspects and potential applications of (ultra) high-pressure homogenisation. Trends Food Sci. Tech..

[19] Liu W., Zhang Z.Q., Liu C.M., Xie M.Y., Liang R.H., Liu J.P., Zou L.Q., Wan J. (2012). Effect of molecular patch modification on the stability of dynamic high-pressure microfluidization treated trypsin. Innov. Food Sci. Emerg..

[20] Iordache M., Jelen P. (2003). High pressure microfluidization treatment of heat denatured whey proteins for improved functionality. Innov. Food Sci. Emerg..

[21] Chen X., Xu X., Zhou G. (2016). Potential of high pressure homogenization to solubilize chicken breast myofibrillar proteins in water. Innov. Food Sci. Emerg..

[22] Dalle Zotte A., Szendro Z. (2011). The role of rabbit meat as functional food. Meat Sci..

[23] FAOSTAT Food and Agriculture Organization Statistics. https://www.fao.org/faostat/en/#home.

[24] Li Z.R., Wang B., Chi C.F., Zhang Q.H., Gong Y.D., Tang J.J., Luo H.Y., Ding G.F. (2013). Isolation and characterization of acid soluble collagens and pepsin soluble collagens from the skin and bone of Spanish mackerel (*Scomberomorous niphonius*). Food Hydrocolloids.

[25] AOAC (2019). Offiicial Methods of Analysis.

[26] Gómez-Guillén M.C., Giménez B., Montero P. (2005). Extraction of gelatin from fish skins by high pressure treatment. Food Hydrocoll..

[27] Ersoy B., Özeren A. (2009). The effect of cooking methods on mineral and vitamin contents of African catfish. Food Chem..

[28] Lowry O.H., Rosebrough N.J., Farr A.L., Randall R.J. (1951). Protein measurement with the folin phenol reagent. J. Biol. Chem..

[29] Herrero A.M., Jiménez-Colmenero F., Carmona P. (2009). Elucidation of structural changes in soy protein isolate upon heating by Raman spectroscopy. Int. J. Food Sci. Tech..

[30] Ullah I., Yin T., Xiong S., Zhang J., Din Z.-U., Zhang M. (2017). Structural characteristics and physicochemical properties of okara (soybean residue) insoluble dietary fiber modified by high-energy wet media milling. LWT-Food Sci. Technol..

[31] Klavons J.A., Bennett R.D. (1987). Nature of the pectin constituent of commercial lemon juice cloud. J. Agric. Food Chem..

[32] Zhang H., Zhao L., Shen Q., Qi L., Jiang S., Guo Y., Zhang C., Richel A. (2021). Preparation of cattle bone collagen peptides-calcium chelate and its structural characterization and stability. LWT-Food Sci. Technol..

[33] Zhang D., Li H., Emara A.M., Hu Y., Wang Z., Wang M., He Z. (2020). Effect of in vitro oxidation on the water retention mechanism of myofibrillar proteins gel from pork muscles. Food Chem..

[34] Zhang D., Li H., Wang Z., Emara A.M., Hu Y., He Z. (2020). Effects of in vitro oxidation on myofibrillar protein charge, aggregation, and structural characteristics. Food Chem..

[35] Etherington D.J., Sims T.J. (1981). Detection and estimation of collagen. J. Sci. Food Agric..

[36] Djabourov M., Lechaire J.P., Gaill F. (1993). Structure and rheology of gelatin and collagen gels. Biorheology.

[37] Paola C.M., Camila A.M., Ana C., Marlon O., Diego S., Robin Z., Beatriz G., Cristina C. (2019). Functional textile finishing of type I collagen isolated from bovine bone for potential healthtech. Heliyon.

[38] Martinez M.G., Bullock A.J., MacNeil S., Rehman I.U. (2019). Characterisation of structural changes in collagen with Raman spectroscopy. Appl. Spectrosc. Rev..

[39] D’Elía N.L., Gravina A.N., Ruso J.M., Laiuppa J.A., Santillán G.E., Messina P.V. (2013). Manipulating the bioactivity of hydroxyapatite nano-rods structured networks: Effects on mineral coating morphology and growth kinetic. Biochim. Biophys. Acta.

[40] Heidari F., Razavi M., Zamani M.A., Tahriri M., Tayebi L. (2018). A comparison between the properties of natural hydroxyapatite produced by cold isostatic pressing and spark plasma sintering techniques. J. Aust. Ceram. Soc..

[41] Nam P.V., Hoa N.V., Trung T.S. (2019). Properties of hydroxyapatites prepared from different fish bones: A comparative study. Ceram. Int..

[42] Guzzi Plepis A.M.D., Goissis G., Das-Gupta D.K. (1996). Dielectric and pyroelectric characterization of anionic and native collagen. Polym. Eng. Sci..

[43] Malde M.K., Bügel S., Kristensen M., Malde K., Graff I.E., Pedersen J.I. (2010). Calcium from salmon and cod bone is well absorbed in young healthy men: A double-blinded randomised crossover design. Nutr. Metab..

[44] Alvarez-Sabatel S., Martinez de Maranon I., Arboleya J.-C. (2015). Impact of high pressure homogenisation (HPH) on inulin gelling properties, stability and development during storage. Food Hydrocolloid..

[45] Leite T.S., Augusto P.E.D., Cristianini M. (2014). The use of high pressure homogenization (HPH) to reduce consistency of concentrated orange juice (COJ). Innov. Food Sci. Emerg..

[46] Chen X., Zou Y., Han M., Pan L., Xing T., Xu X., Zhou G. (2016). Solubilisation of myosin in a solution of low ionic strength L-histidine: Significance of the imidazole ring. Food Chem..

[47] Hayakawa T., Ito T., Wakamatsu J., Nishimura T., Hattori A. (2009). Myosin is solubilized in a neutral and low ionic strength solution containing L-histidine. Meat Sci..

[48] Omana D.A., Xu Y., Moayedi V., Betti M. (2010). Alkali-aided protein extraction from chicken dark meat: Chemical and functional properties of recovered proteins. Process Biochem..

[49] Asserin J., Lati E., Shioya T., Prawitt J. (2015). The effect of oral collagen peptide supplementation on skin moisture and the dermal collagen network: Evidence from an ex vivo model and randomized, placebo-controlled clinical trials. J. Cosmet. Dermatol..

[50] De Luca C., Mikhal’chik E.V., Suprun M.V., Papacharalambous M., Truhanov A.I., Korkina L.G. (2016). Skin antiageing and systemic redox effects of supplementation with marine collagen peptides and plant-derived antioxidants: A single-blind case-control clinical study. Oxid. Med. Cell. Longev..

[51] Kimira Y., Ogura K., Taniuchi Y., Kataoka A., Inoue N., Sugihara F., Nakatani S., Shimizu J., Wada M., Mano H. (2014). Collagen-derived dipeptide prolyl-hydroxyproline promotes differentiation of MC3T3-E1 osteoblastic cells. Biochem. Biophys. Res. Commun..

[52] Park S.H., Jo Y.J. (2019). Static hydrothermal processing and fractionation for production of a collagen peptide with anti-oxidative and anti-aging properties. Process Biochem..

[53] Zdzieblik D., Oesser S., Gollhofer A., Koenig D. (2017). Improvement of activity-related knee joint discomfort following supplementation of specific collagen peptides. Appl. Physiol. Nutr. Metab..

[54] Elam M.L., Johnson S.A., Hooshmand S., Feresin R.G., Arjmandi B.H. (2015). A Calcium-Collagen chelate dietary supplement attenuates bone loss in postmenopausal women with osteopenia: A randomized controlled trial. J. Med. Food.

[55] Guo L., Harnedy P.A., O’Keeffe M.B., Zhang L., Li B., Hou H., FitzGerald R.J. (2015). Fractionation and identification of Alaska pollock skin collagen-derived mineral chelating peptides. Food Chem..

[56] Guo H., Hong Z., Yi R. (2015). Core-shell collagen peptide chelated calcium/calcium alginate nanoparticles from fish scales for calcium Supplementation. J. Food Sci..

[57] Song X., Zhou C., Fu F., Chen Z., Wu Q. (2013). Effect of high-pressure homogenization on particle size and film properties of soy protein isolate. Ind. Crop. Prod..

[58] Stani C., Vaccari L., Mitri E., Birarda G. (2020). FTIR investigation of the secondary structure of type I collagen: New insight into the amide III band. Spectrochim. Acta A.

[59] Barth A., Zscherp C. (2002). What vibrations tell us about proteins. Q. Rev. Biophys..

[60] Herrero A.M. (2008). Raman spectroscopy a promising technique for quality assessment of meat and fish: A review. Food Chem..

[61] Li-Chan E.C.Y. (1996). The applications of Raman spectroscopy in food science. Trends Food Sci. Tech..

[62] Thawornchinsombut S., Park J.W., Meng G.T., Li-Chan E.C.Y. (2006). Raman spectroscopy determines structural changes associated with gelation properties of fish proteins recovered at alkaline pH. J. Agric. Food Chem..

[63] Zhang Z., Yang Y., Zhou P., Zhang X., Wang J. (2017). Effects of high pressure modification on conformation and gelation properties of myofibrillar protein. Food Chem..

[64] Peng H.L., Chen S., Luo M., Ning F.J., Zhu X.M., Xiong H. (2016). Preparation and self-assembly mechanism of bovine serum albumin-citrus peel pectin conjugated hydrogel: A potential delivery system for vitamin C. J. Agric. Food Chem..

[65] Zhang Y.P., Yang R.J., Zhang W.N., Hu Z.X., Zhao W. (2017). Structural characterization and physicochemical properties of protein extracted from soybean meal assisted by steam flash-explosion with dilute acid soaking. Food Chem..

[66] Yongsawatdigul J., Sinsuwan S. (2007). Aggregation and conformational changes of tilapia actomyosin as affected by calcium ion during setting. Food Hydrocolloid..

[67] Gao Y., Wang L., Qiu Y., Fan X., Zhang L., Yu Q.L. (2022). Valorization of cattle slaughtering industry by-products: Modification of the functional properties and structural characteristics of cowhide gelatin induced by high hydrostatic pressure. Gels.

[68] Guo Z., Huang Z., Guo Y., Li B., Yu W., Zhou L., Jiang L., Teng F., Wang Z. (2021). Effects of high-pressure homogenization on structural and emulsifying properties of thermally soluble aggregated kidney bean (*Phaseolus vulgaris* L.) proteins. Food Hydrocoll..

[69] Liu H.-H., Kuo M.-I. (2016). Ultra high pressure homogenization effect on the proteins in soy flour. Food Hydrocolloid..

[70] McKerchar H.J., Clerens S., Dobson R.C.J., Dyer J.M., Maes E., Gerrard J.A. (2019). Protein-protein crosslinking in food: Proteomic characterisation methods, consequences and applications. Trends Food Sci. Tech..

[71] Arfat Y.A., Benjakul S. (2012). Impact of zinc salts on heat-induced aggregation of natural actomyosin from yellow stripe trevally. Food Chem..

[72] Lund M.N., Heinonen M., Baron C.P., Estévez M. (2011). Protein oxidation in muscle foods: A review. Mol. Nutr. Food Res..

[73] Du X., Sun Y., Pan D., Wang Y., Ou C., Cao J. (2018). Change of the structure and the digestibility of myofibrillar proteins in Nanjing dry-cured duck during processing. J. Sci. Food. Agric..

[74] Diemer V., Ollivier N., Leclercq B., Drobecq H., Vicogne J., Agouridas V., Melnyk O. (2020). A cysteine selenosulfide redox switch for protein chemical synthesis. Nat. Commun..

[75] Zhuang X., Han M., Jiang X., Bai Y., Zhou H., Li C., Xu X.-L., Zhou G.-H. (2019). The effects of insoluble dietary fiber on myofibrillar protein gelation: Microstructure and molecular conformations. Food Chem..

